# Increased Blood-Reelin-Levels in First Episode Schizophrenia

**DOI:** 10.1371/journal.pone.0134671

**Published:** 2015-08-25

**Authors:** Tobias Hornig, Lukas Sturm, Bernd Fiebich, Ludger Tebartz van Elst

**Affiliations:** Department of Psychiatry, Albert-Ludwigs-University, Hauptstr. 5, 79104 Freiburg, Germany; The Nathan Kline Institute, UNITED STATES

## Abstract

**Background:**

Reelin is an extracellular glycoprotein involved in several functions of brain development, synaptogenesis and dendritic proliferation. Numerous studies found perturbation in the reelin system and altered serum reelin levels in neuropsychiatric patients using the western blot procedure. In the international literature, this is the first study that made use of an enzyme-linked immunosorbent assay to analyze serum reelin protein concentration quantitatively.

**Rationale:**

In order to study possible alterations in reelin blood levels in schizophrenia, we analyzed this signal in schizophrenic patients with a first episode hallucinatory and paranoid syndrome and control subjects in a pilot study design.

**Results:**

We found increased blood reelin protein concentration in schizophrenic patients compared to healthy controls.

**Discussion:**

Our findings point to a relevant role of reelin metabolism in the pathogenesis of schizophrenia.Reelin could be a biomarker for the course of disease or psychopharmacological treatment.

**Conclusion:**

We conclude that the reelin protein blood concentration might be a relevant signal with respect to the pathophysiology of schizophrenia.

## Introduction

Reelin (RELN) is a large extracellular glycoprotein involved in embryonic neuronal migration and in several functions of brain development [1–3]. It is expressed by CajalRetzius cells in layer I and GABAergic interneurons in all layers of the cortex and hippocampus as well as in the cerebellum by glutamatergic neurons [4,5]. At present five processed fragments in biopsy fluids (340-kDa, 270-kDa, 190-kDa, 180-kDa, 80-kDa) of this 450 kDa large protein are known[6,7]. Furthermore, RELN plays an important role in dendritic growth, synaptic plasticity and, as a modulatory element of the N-Methyl-D-Aspartate (NMDA) receptor [8,9], in long-term potentiation (LTP) and thus also in memory and learning [10]. In line with these findings Apo- E- and Very low-density lipoprotein (VLDL)- receptor deficient knockout mice respectively display an impairment in fear conditioned memory formation, spatial learning and defective LTP induction [11–13]. Disturbed RELN metabolism leads to deficient cortical lamination for example in the Reeler Mouse phenotype[2,14–16]or to lissencephaly in humans [17,18]. Lissencephaly is a condition in which the cerebral and cerebellar gyration is reversed with very different characteristics like mental retardation, epilepsy or muscle spasticity [2,19–21]. Also, there is evidence that RELN could be involved in other neuropsychiatric disorders such as Alzheimer’s disease, frontotemporal dementia, autism spectrum disorders, bipolar affective disorders, some subtypes of epilepsy and also schizophrenia[22–27].

### RELN-findings in schizophrenia

Table 1 summarized all RELN-related findings with respect to schizophrenia.

**Table 1:** Summary of RELN-related findings in schizophrenia.

Study	Type of study	SampleSize S/C	RELN mRNA Conc.	RELN Blood Conc.	mRNA DNMT1 in GCN	Findings
Fatemi et al. 2005	Post mortem study of brain tissue	15/15	↓CBL	n/a	n/a	p<0.05 vs. controls
Habl et al. 2012	Post mortem study of brain tissue	12/13	↓PFC, left	n/a	n/a	p = 0.022 vs. controls in white matter
						p = 0.007 vs. controls in gray matter
Impagnatiello et al. 1998	Post mortem study of brain tissue	18/18	↓PFC, HIP, CN, CBL	n/a	n/a	p<0.02 vs. controls
Giodotti et al. 2000	Post mortem study of brain tissue	15/15	↓PFC, CBL	n/a	n/a	p<0.001 vs. controls in PFC
						p = 0.02 vs. controls in cerebellum
Zhubi et. al 2009	Post mortem study of brain tissue	6/7	n/a	n/a	↑ PFC	p = 0.007 vs. controls
Zhubi et. al 2009	Analysis of blood cells **in vivo**	22/24	n/a	n/a	↑ PBL	p = 0.001 vs. controls
Giodotti et al. 2007	Post mortem study of brain tissue	14/15	n/a	n/a	↑PFC	p = 0.005 vs. controls
Veldic et al. 2005	Post mortem study of brain tissue	19/26	n/a	n/a	↑PFC	p<0.001 vs. controls
Veldic et al. 2004	Post mortem study of brain tissue	7/8		n/a	↑PFC	p = 0.025 vs. controls
			↓PFC			p<0.05 vs. controls
Fatemi et al. 2001	Analysis of blood RELN level **in vivo**	16/8	n/a	↑	n/a	p = 0.02 vs. controls for 410 kDa F.

[Altered Reelin levels in blood and brain tissue in schizophrenic patients (S) vs. healthy controls (C); Conc = Concentration, GCN = Gabaergic Cerebral (Inter-) Neuron, PFC = Prefrontal Cortex, HIP = Hipocampus, CN = caudate nucleus, CBL = Cerebellum, PBL = Peripheral Blood Lymphocytes

↓ = decrease

↑ = increase, n/a = not applicable].

Post mortem analyses revealed reduced RELN mRNA in the brains of schizophrenic patients [28–31]. Especially in the prefrontal lobe and hippocampus as well as in the cerebellum, a smaller dendritic length and a reduced density of dendritic spines were found [1,30,32,33]. Fatemi et al. found increased blood RELN concentration in patients with schizophrenia and decreased RELN concentration in bipolar affective disorders [34]. Others show an increased cerebrospinal RELN concentration in patients with Alzheimers disease and Frontotemporal Dementia [23,35]. Current studies indicate hypermethylation of the RELN- gene promotor in patients with schizophreniform and autism spectrum disorders while the DNA Methyltransferase 1 (DNMT1) mRNA concentration was increased in the same samples, whereby DNMT1 methylates the RELN promotor.[36–39]. These results point to a possible pathogenetic effect of RELN in different neuropsychiatric disorders. Some drugs, such as the anticonvulsant and mood stabilizer valproic acid, cause a demethylation of these regions and a acetylation of histone proteins and therefore increased transcription rates of the RELN gene [29,40,41].

In the past, schizophrenia was thought to be a degenerative development disorder [42,43]. In the international literature, however, there are neither clues for cortical dyslamination such as in the reeler mouse or in patients with lissencephaly nor convincing evidence for morphological neurodegenerative aspects [44,45].Nevertheless there are a lot of hints for synaptic dysfunction and lack of neuronal plasticity [46–48].

As aforementioned, a lack of higher cognitive functions and neuronal plasticity is assumed to be caused by defective RELN homoeostasis. Numerous previous studies found respective deficits in schizophrenic patients especially in verbal and working memory, spatial learning as well as executive functions[49–53]. On the basis of this findings and considering the preceding studies we hypothesized altered blood RELN concentration in schizophrenia without prediction the direction of alteration.

To test this hypothesis and to get quantitative figures of blood reelin protein concentration in schizophrenic and healthy subjects, we used an Enzyme-linked immunosorbent assay (ELISA) of serum RELN-protein. This approach is unprecedented so far and for that reason no standard values were defined until now.

## Materials and Methods

### 2.1 Patients and control subjects

Twenty medicated patients with Diagnostic and Statistical Manual of Mental Disease (DSM)-IV-defined schizophrenia (10 male and 10 female), hospitalized for acute exacerbation were identified among the in-patients of the Department of Psychiatry and Psychotherapy of the University Hospital of Freiburg which were treated unsolicited and voluntarily. The clinical diagnosis of schizophrenia was made by experienced senior consultant psychiatrists based on a structured interview according to DSM-IV criteria, the ability of consent based on the psychopathological interview according to the Arbeitsgemeinschaftfür Methodik und Dokumentation in der Psychiatrie (AMDP)-system and refers to the common principles of decision-making[54]. After having given informed written consent to participate on a voluntary basis, the patients were included into this study. We did not include patients that where housed judicial or where situated in legal care. All patients received a full neurological and medical history and examination as well as routine blood tests and EEG examinations in order to exclude any other medical or neurological disorder. Patients were excluded if they had any other DSM-IV axis I diagnosis or met criteria for substance abuse within the previous 6 months. In order to keep the sample homogenous by avoiding an overlap of psychogeriatric problems, the study groupwas restricted to patients with a first episode diagnosis of schizophrenia under the age of 45 years and with a normal IQ range. The control subjects were recruited in our neurological ambulance where subjects were assessed by experienced senior consultants. Based on a comprehensive neuropsychiatric assessment including a thorough medical and family possible all neuropsychiatric disorders were excluded.

#### 2.1.1 Ethics Statement

Approval from the Albert-Ludwigs University ethics committee Freiburg was obtained before onset of the study (no.: 335/13).

### 2.2 Enzyme-Linked Immunosorbent Assay

We used an ELISA kit by Cusabio, performing a quantitative sandwich enzyme immunoassay that detects human reelin-protein in serum, plasma and tissue homogenates. Its detection range is 0.156 ng/μl–10 ng/μl, the sensitivity is typically <0.039 ng/μl. Sample signals below the detectable concentration limit were therefore approximated 0 ng/μl. The assay is highly specific for human reelin. Repeated testing of reference samples accounted for a recovery rate of 94,59% in human serum. No significant cross reactivies have been observed. Intra- and inter-assay coefficients of variation, determined by testing three reference samples twenty times on one plate respectively on twenty different plates, are<8% respectively <10%. Thus retest reliability of this method is very good. Epitope mapping was not done.

The standard series was obtained by performing six 1:2 dilution steps starting from a high standard with an antigen concentration of 10 ng/ml (thus resulting in concentrations of 5 ng/ml, 2.5 ng/ml, 1.25 ng/ml, 0.625 ng/ml, 0.312 ng/ml, 0.156 ng/ml). A solution with a concentration of 0 ng/ml served as zero standard.

100 μl of sample respectively standard at a time were pipetted into the wells of a 12 x 8 polystyrene microtiter plate precoated with a human reelin-specific monoclonal mouse antibody and incubated for two hours at 37°C. After removal of supernatant 100 μl of a solution containing biotinylated polyclonal mouse antibody specific for human reelin was added to each well and incubated for one hour at 37°C. Unbound detection antibody was thereafter removed by applying 200 μl of wash buffer and aspirating three times per well. Subsequently 100 μl of avidin-bound HRP (horseradish peroxidase) solution were added to each well and incubated for one hour at 37°C. Afterwards aspiration/washing was repeated five times. Then 90 μl of TMB substrate (3.3',5.5'-tetramethylebenzidine) were added and incubated at 37°C. The probes were protected from light. After 25 minutes 50 μl of stop solution (1N sulfuric acid) at a time were added. Within a period of five minutes the optical densities were determined using a Dynex MRX microplate reader set to a wavelength of 450 nm. Samples and standards were then evaluated with the Dynex Revelations software.

#### 2.2.1 Western Blot

For electrophoresis, equal amounts of serum (3 μl) were loaded per gel lane, size-fractionated on a 3–8% Tris-acetate-polyacrylamide gel (Invitrogen, Karlsruhe, Germany), and blotted onto a polyvinylidenediflouride (PVDF) membrane (Roche, Mannheim, Germany). For immunodetection, PVDF membranes were pretreated with I-Block buffer (Tropix, Bedford, MA, USA) followed by incubation with the following antibodies: mouse monoclonal anti-human Reelin (1:500; 142; Chemicon) or rabbit polyclonal anti-Ceruloplasmin (1:5000; Dako, Glostrup, Denmark) for 1 h at room temperature. After several washing steps, the membranes were incubated with the respective alkaline-phosphatase-conjugated secondary antibody (1:10.000; Tropix) for 1 h at room temperature and CDP Star (Tropix) was used as substrate for chemiluminescent detection by the *Chemismart* System (Peqlab Biotechnologies, Erlangen, Germany).

Quantification of Western blot signals was performed by optical density (OD) measurement using the Bio-1D software (Peqlab). OD values were determined for each of the three Reelin isoforms (400, 320 and 180 kDa) detected by the Reelin antibody. Data were normalized to Ceruloplasmin signals as loading control.

### 2.3 Statistical Analysis

We analyzed the socio-demographically comparability of our patient and control group using a T-test for metric variables like age and a Chi-square-test for categorical variables like gender or education. Between group effects in our primary outcome measure was analyzed using a standard t-test procedure with group as main factor and RELN concentration as depending variable. In order to analyse a possible confounding effect of the factor medication on RELN signal we compared RELN levels between the different main antipsychotic groups (risperdone: n = 12; olanzapine: n = 5; aripiprazole: n = 1). In order to analyse a possible interaction between factors like school education, or family history for psychosis or any other psychiatric disorder we additionally calculated an univariate ANOVA in a general linear model with these factors. A p-value of .05 was chosen as the criterion of significance.

## Results

### 3.1 Demographic details

Table 2 summarizes the demographic and psychosocial data of our patients and group matched healthy controls. Two probands had to be excluded from both groups because the samples’ reelin concentrations were outside the detection range:

Both groups were matched in terms of age, gender and school education.

**Table 2:** Demographic data and RELN levels of our patients and group matched healthy controls.

	Schiz. (n = 18)	Contr. (n = 18)	Statistics
**Age**	26.22 [5.07]*	26.33 [3.94]*	T = -0.073; df = 34, p = .94
**Gender**	F:M / 8:10	F:M / 9:9	Chi^2^ = 0.22, df = 1, p = 0.896
**School education**	L:M:H / 3:7:8	L:M:H / 4:5:9	Chi^2^ = 0.11, df = 1, p = 0.5
**RELN conc.** ^1^	2.59 ng/μl [1,5]*	1.32 ng/μl [1,1]*	T = 2,8, df = 34, p = .007
**RELN conc.** ^2^	0.587 ng/μl [0.582]*	0.367 ng/μl [0.287]*	T = 1.43, df = 34, p = .16

[Age/RELN conc.:

*mean [SD]; Gender: F = female, M = male; School education: L = low, M = middle, H = high; conc. = concentration, conc.

^1^ with ELISA, conc.

^2^with Western Blot].

### 3.2 Serum reelin concentration

**Fig 1:**
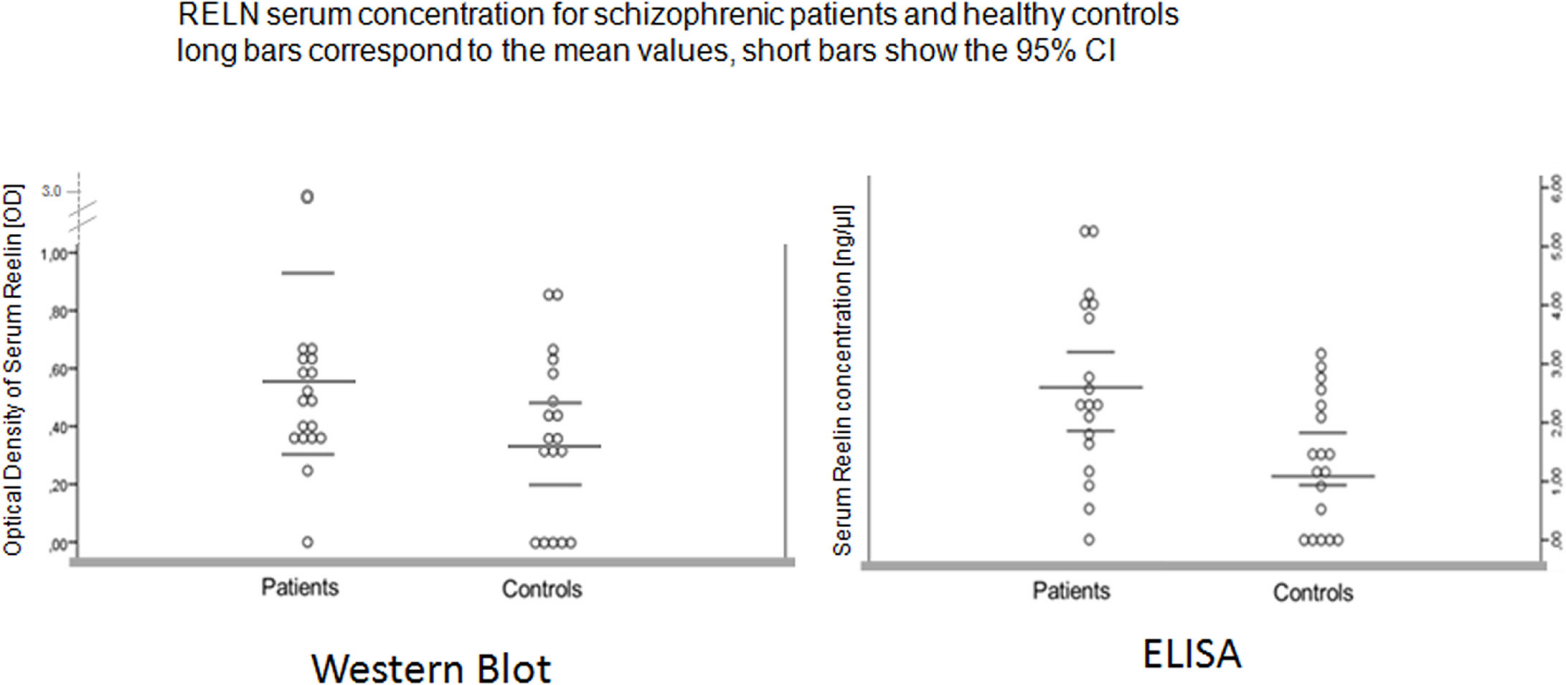
RELN serum concentration for schizophrenic patients and healthy controls. Long bars correspond to the mean values, short bars show the 95% Cl.

We found a mean serum RELN concentration of 1.32 ng/μl on the side of the healthy controls as can be seen in Table 2. The range was from under 0.156 ng/μl until 3.17 ng/μl. In comparison the mean serum RELN concentration of patients was 2.59 ng/μl and thus nearly twice as large as the concentration of controls with a range from under 0.156 ng/μl until 5.29 ng/μl (see Fig 1). Thus, five patients were out of the range of the healthy controls.

#### 3.2.1 Group comparison concerning serum reelin levels

Table 2 also shows that the mean value of the serum RELN concentration in patients and healthy controls differed significantly in that patients displayed significantly increased signals (p = 0.007, df = 34, T = 2.8). Fig 1 illustrates the scatterplot of this main finding of our pilot study. There was no significant interaction between RELN signals and school education (p = 0.314, F = 1,2), medication (p = 0.581, F = 0.56), family history of mental illness (p = 0.477, F = 0,51), age (p = 0.179, F = 1.89) or gender (p = 0.834, F = 0.04) in the two groups.

## Discussion

In this pilot study we measured the blood RELN protein concentration of schizophrenic patients and healthy controls with an Enzyme-linked immunosorbent assay.

Our main finding was an increased blood RELN protein level in schizophrenic patients. This finding relates well to the only other study that analyzed RELN blood levels i.e. the paper byFatemi et al. who also found increased blood RELN concentrations in schizophrenic patients compared to healthy controls [34]. Apart from that there are a number of studies analyzing RELN and DNA Methyltransferase 1 (DNMT1) mRNA concentration in the cerebrum and cerebellum (Table 1).

To compare our finding obtained with the ELISA method to findings of other authors who used the western blot procedure in an post-hoc approach we also measured the serum RELN protein concentration with the Western Blot technique following Tinnes S, Ringwald J and Haas CA [55]. For that purpose we only included those samples with signals above the predefined ELISA cut off threshold of 0.156 ng/μl. Doing this we found a significant correlation between the ELISA and Western Blot signals for the 340 k-Da RELN fragment (r = 0.4, p = 0.02). Comparing Western Blot based RELN signals between groups we again identified increased average RELN levels in schizophrenic patients (0.58 Optical Density) vs. controls (0,36 Optical Density), which just failed to reach level of significance (p = 0.16, T = 1.4) as you can see in Fig 1. This is no surprise given the small sample size and corresponds well to the findings of Fatemi et al. in Caucasian schizophrenics [34].

When also including those patients with signals below the official ELISA detection threshold, there was no significant correlation between the two signals. This of course reflects the fact that including measurement outside the accepted measurement range of a method nessecarily induces noise in each experiment. Obviously the discriminating power of ELISA-based RELN signal detection below the recommended threshold should be regarded with caution.

Presently, we do not know how increased serum RELN signals translate into cerebral RELN concentrations. Up to date only little is known about the mutual interaction between peripheral and brain RELN homeostasis. Apart from the RELN expression in cerebral cells, there are clues for its expression in liver-, plasma-, adrenal- and pituitary cells with a different glycosylation in plasma and cerebral fluid [35,56,57]. Moreover, there are several different influences such as post translational effects, loss of origin cells (e.g. GABAergic interneurons) or medication that could have variable or even opposite effects on the concentration of RELN in cerebral spinal fluid (CSF) or blood respectively[58]. Therefore conclusions about the interaction between peripheral and central RELN levels should be handled carefully.

Until now, only three chronically schizophrenic patients were measured with regard to the RELN concentration of cerebral fluid in vivo [59]. At present, there is no empirical information about the dynamics of RELN levels in the brain or cerebral fluid over the course of disease or in relation to healthy controls.

However, there is evidence to suggest that reelin (N-terminal 180 kDa fragment) regulates dendritic maturation in vitro [60]. Blocked serotonin receptors (5-HT 3A subtype) in CajalRetzius cells caused a reduced reelin release and a dysregulated dendritic proliferation. A similarly altered dendritic complexity was found in 5-HT 3A receptor lacking mice [61,62]. On the other hand the expression of 5-HT 3A receptors in peripheral blood mononuclear cells (lymphocytes) was up-regulated after medication with antipsychotic drugs [63] and studies with serotonin transporter antagonists indicate higher serum RELN levels in adults [64]. Therefore we have to consider that our finding might be modulated by medication effects. If this was the case RELN concentration could serve as an objective marker of therapeutic response to neuroleptic drugs. Supporting such assumptions there is preliminary evidence that some psychopharmacological drugs are involved in epigenetic mechanisms of the RELN promoter region and enhance the transcription rate of RELN[59]. All of these findings are in line with previous findings and indicate, that a psychopharmacological therapy could affect the peripheral RELN homoeostasis [29,38,40,41,65]. However, further research is nessecary to prove such propositions.

The possible meaning of our finding: Taking together all available data and our findings there is ample evidence to support the notion that the RELN system might well play an important role in the pathophysiology of schizophrenia. It might even turn out to be a possible biomarker for the course of disease. Thus, in relationship to other findings, RELN could indicate the response of psychopharmacological medication [56]. At present it is unknown how the altered expression of RELN in brain or peripheral tissue is regulated in the course of disease irrespective of psychopharmacological medication. This should be subject of further studies.

Methodological limitation: The sample size of our study is small but comparable to similar studies. Furthermore we did not measure unmedicated patients and there was no parallel assessment of peripheral and central RELN. Altogether we do not know actually how far we measured a medical effect or how far cerebral or peripheral mechanisms are responsible for blood or CSF RELN levels in the course of disease.

Furthermore no standard values in relation to the serum RELN concentration were defined. So we cannot validate our results or compare it with results in the international literature. However, this is not a critical point at this stage of research, where case-control studies are the method of choice to increase our knowledge of this interesting signal.

Summary: We found an increased level of blood RELN concentration in patients with schizophrenia in comparison to a healthy control population. Our finding is in line with the only other study analyzing blood RELN signal in schizophrenia. Further studies with larger sample size will have to answer the question in which way the RELN concentration is related to the dynamics of a psychopharmacological therapy respectively the blood levels of the psychopharmacological agents and in which way the cerebral and peripheral RELN levels are linked to the schizophreniform disease processes.

## Supporting Information

S1 Dataset (PDF)

## References

[1] Del Río JA, Heimrich B, Borrell V, Förster E, Drakew A, Alcántara S, et al A role for Cajal-Retzius cells and reelin in the development of hippocampal connections. Nature. 1997;385: 70–74. 10.1038/385070a0 8985248

[2] Hoffarth RM, Johnston JG, Krushel LA, van der Kooy D. The mouse mutation reeler causes increased adhesion within a subpopulation of early postmitotic cortical neurons. J Neurosci Off J Soc Neurosci. 1995;15: 4838–4850. 10.1523/JNEUROSCI.15-07-04838.1995 PMC6577875 7623115

[3] Caviness VS, Rakic P. Mechanisms of cortical development: a view from mutations in mice. Annu Rev Neurosci. 1978;1: 297–326. 10.1146/annurev.ne.01.030178.001501 386903

[4] Alcántara S, Ruiz M, D’Arcangelo G, Ezan F, de Lecea L, Curran T, et al Regional and cellular patterns of reelin mRNA expression in the forebrain of the developing and adult mouse. J Neurosci Off J Soc Neurosci. 1998;18: 7779–7799. 10.1523/JNEUROSCI.18-19-07779.1998 PMC6792998 9742148

[5] Pesold C, Impagnatiello F, Pisu MG, Uzunov DP, Costa E, Guidotti A, et al Reelin is preferentially expressed in neurons synthesizing gamma-aminobutyric acid in cortex and hippocampus of adult rats. Proc Natl Acad Sci U S A. 1998;95: 3221–3226. 9501244 10.1073/pnas.95.6.3221 PMC19723

[6] Lambert de Rouvroit C, de Bergeyck V, Cortvrindt C, Bar I, Eeckhout Y, Goffinet AM. Reelin, the extracellular matrix protein deficient in reeler mutant mice, is processed by a metalloproteinase. Exp Neurol. 1999;156: 214–217. 10.1006/exnr.1998.7007 10192793

[7] Jossin Y. Neuronal migration and the role of reelin during early development of the cerebral cortex. Mol Neurobiol. 2004;30: 225–251. 10.1385/MN:30:3:225 15655250

[8] Teixeira CM, Kron MM, Masachs N, Zhang H, Lagace DC, Martinez A, et al Cell-autonomous inactivation of the reelin pathway impairs adult neurogenesis in the hippocampus. J Neurosci Off J Soc Neurosci. 2012;32: 12051–12065. 10.1523/JNEUROSCI.1857-12.2012 PMC3475414 22933789

[9] Finkbeiner S, Tavazoie SF, Maloratsky A, Jacobs KM, Harris KM, Greenberg ME. CREB: a major mediator of neuronal neurotrophin responses. Neuron. 1997;19: 1031–1047. 9390517 10.1016/s0896-6273(00)80395-5

[10] Rogers JT, Zhao L, Trotter JH, Rusiana I, Peters MM, Li Q, et al Reelin supplementation recovers sensorimotor gating, synaptic plasticity and associative learning deficits in the heterozygous reeler mouse. J Psychopharmacol Oxf Engl. 2013;27: 386–395. 10.1177/0269881112463468 PMC3820099 23104248

[11] Barr AM, MacLaurin SA, Semenova S, Fish KN, Markou A. Altered performance of reelin-receptor ApoER2 deficient mice on spatial tasks using the Barnes maze. Behav Neurosci. 2007;121: 1101–1105. 10.1037/0735-7044.121.5.1101 17907841

[12] Beffert U, Weeber EJ, Durudas A, Qiu S, Masiulis I, Sweatt JD, et al Modulation of synaptic plasticity and memory by Reelin involves differential splicing of the lipoprotein receptor Apoer2. Neuron. 2005;47: 567–579. 10.1016/j.neuron.2005.07.007 16102539

[13] Weeber EJ, Beffert U, Jones C, Christian JM, Forster E, Sweatt JD, et al Reelin and ApoE receptors cooperate to enhance hippocampal synaptic plasticity and learning. J Biol Chem. 2002;277: 39944–39952. 10.1074/jbc.M205147200 12167620

[14] D’Arcangelo G, Miao GG, Chen SC, Soares HD, Morgan JI, Curran T. A protein related to extracellular matrix proteins deleted in the mouse mutant reeler. Nature. 1995;374: 719–723. 10.1038/374719a0 7715726

[15] Ogawa M, Miyata T, Nakajima K, Yagyu K, Seike M, Ikenaka K, et al The reeler gene-associated antigen on Cajal-Retzius neurons is a crucial molecule for laminar organization of cortical neurons. Neuron. 1995;14: 899–912. 7748558 10.1016/0896-6273(95)90329-1

[16] Rakic P. Principles of neural cell migration. Experientia. 1990;46: 882–891. 2209797 10.1007/BF01939380

[17] Saillour Y, Carion N, Quelin C, Leger P-L, Boddaert N, Elie C, et al LIS1-related isolated lissencephaly: spectrum of mutations and relationships with malformation severity. Arch Neurol. 2009;66: 1007–1015. 10.1001/archneurol.2009.149 19667223

[18] Willemsen MH, Vissers LEL, Willemsen MAAP, van Bon BWM, Kroes T, de Ligt J, et al Mutations in DYNC1H1 cause severe intellectual disability with neuronal migration defects. J Med Genet. 2012;49: 179–183. 10.1136/jmedgenet-2011-100542 22368300

[19] Merwick A, O’Brien M, Delanty N. Complex single gene disorders and epilepsy. Epilepsia. 2012;53 Suppl 4: 81–91. 10.1111/j.1528-1167.2012.03617.x 22946725

[20] Poduri A, Evrony GD, Cai X, Walsh CA. Somatic mutation, genomic variation, and neurological disease. Science. 2013;341: 1237758 10.1126/science.1237758 23828942 PMC3909954

[21] Sohal APS, Montgomery T, Mitra D, Ramesh V. TUBA1A mutation-associated lissencephaly: case report and review of the literature. Pediatr Neurol. 2012;46: 127–131. 10.1016/j.pediatrneurol.2011.11.017 22264709

[22] Liu JS. Molecular genetics of neuronal migration disorders. Curr Neurol Neurosci Rep. 2011;11: 171–178. 10.1007/s11910-010-0176-5 21222180

[23] Saez-Valero J, Costell M, Sjögren M, Andreasen N, Blennow K, Luque JM. Altered levels of cerebrospinal fluid reelin in frontotemporal dementia and Alzheimer’s disease. J Neurosci Res. 2003;72: 132–136. 10.1002/jnr.10554 12645087

[24] Knuesel I. Reelin-mediated signaling in neuropsychiatric and neurodegenerative diseases. Prog Neurobiol. 2010;91: 257–274. 10.1016/j.pneurobio.2010.04.002 20417248

[25] Kelemenova S, Ostatnikova D. Neuroendocrine pathways altered in autism. Special role of reelin. Neuro Endocrinol Lett. 2009;30: 429–436. 20010491

[26] Fatemi SH. Reelin glycoprotein: structure, biology and roles in health and disease. Mol Psychiatry. 2005;10: 251–257. 10.1038/sj.mp.4001613 15583703

[27] Fatemi SH, Hossein Fatemi S, Stary JM, Earle JA, Araghi-Niknam M, Eagan E. GABAergic dysfunction in schizophrenia and mood disorders as reflected by decreased levels of glutamic acid decarboxylase 65 and 67 kDa and Reelin proteins in cerebellum. Schizophr Res. 2005;72: 109–122. 10.1016/j.schres.2004.02.017 15560956

[28] Habl G, Schmitt A, Zink M, von Wilmsdorff M, Yeganeh-Doost P, Jatzko A, et al Decreased reelin expression in the left prefrontal cortex (BA9) in chronic schizophrenia patients. Neuropsychobiology. 2012;66: 57–62. 10.1159/000337129 22797278

[29] Chen Y, Sharma RP, Costa RH, Costa E, Grayson DR. On the epigenetic regulation of the human reelin promoter. Nucleic Acids Res. 2002;30: 2930–2939. 12087179 10.1093/nar/gkf401 PMC117056

[30] Impagnatiello F, Guidotti AR, Pesold C, Dwivedi Y, Caruncho H, Pisu MG, et al A decrease of reelin expression as a putative vulnerability factor in schizophrenia. Proc Natl Acad Sci U S A. 1998;95: 15718–15723. 9861036 10.1073/pnas.95.26.15718 PMC28110

[31] Guidotti A, Auta J, Davis JM, Di-Giorgi-Gerevini V, Dwivedi Y, Grayson DR, et al Decrease in reelin and glutamic acid decarboxylase67 (GAD67) expression in schizophrenia and bipolar disorder: a postmortem brain study. Arch Gen Psychiatry. 2000;57: 1061–1069. 11074872 10.1001/archpsyc.57.11.1061

[32] Niu S, Renfro A, Quattrocchi CC, Sheldon M, D’Arcangelo G. Reelin promotes hippocampal dendrite development through the VLDLR/ApoER2-Dab1 pathway. Neuron. 2004;41: 71–84. 14715136 10.1016/s0896-6273(03)00819-5

[33] Glantz LA, Lewis DA. Decreased dendritic spine density on prefrontal cortical pyramidal neurons in schizophrenia. Arch Gen Psychiatry. 2000;57: 65–73. 10632234 10.1001/archpsyc.57.1.65

[34] Fatemi SH, Kroll JL, Stary JM. Altered levels of Reelin and its isoforms in schizophrenia and mood disorders. Neuroreport. 2001;12: 3209–3215. 11711858 10.1097/00001756-200110290-00014

[35] Botella-López A, Burgaya F, Gavín R, García-Ayllón MS, Gómez-Tortosa E, Peña-Casanova J, et al Reelin expression and glycosylation patterns are altered in Alzheimer’s disease. Proc Natl Acad Sci U S A. 2006;103: 5573–5578. 10.1073/pnas.0601279103 16567613 PMC1414634

[36] Roth TL, Lubin FD, Sodhi M, Kleinman JE. Epigenetic mechanisms in schizophrenia. Biochim Biophys Acta. 2009;1790: 869–877. 10.1016/j.bbagen.2009.06.009 19559755 PMC2779706

[37] Guidotti A, Ruzicka W, Grayson DR, Veldic M, Pinna G, Davis JM, et al S-adenosyl methionine and DNA methyltransferase-1 mRNA overexpression in psychosis. Neuroreport. 2007;18: 57–60. 10.1097/WNR.0b013e32800fefd7 17259861

[38] Grayson DR, Chen Y, Costa E, Dong E, Guidotti A, Kundakovic M, et al The human reelin gene: transcription factors (+), repressors (-) and the methylation switch (+/-) in schizophrenia. Pharmacol Ther. 2006;111: 272–286. 10.1016/j.pharmthera.2005.01.007 16574235

[39] Veldic M, Guidotti A, Maloku E, Davis JM, Costa E. In psychosis, cortical interneurons overexpress DNA-methyltransferase 1. Proc Natl Acad Sci U S A. 2005;102: 2152–2157. 10.1073/pnas.0409665102 15684088 PMC548582

[40] Tremolizzo L, Doueiri M-S, Dong E, Grayson DR, Davis J, Pinna G, et al Valproate corrects the schizophrenia-like epigenetic behavioral modifications induced by methionine in mice. Biol Psychiatry. 2005;57: 500–509. 10.1016/j.biopsych.2004.11.046 15737665

[41] Dong E, Nelson M, Grayson DR, Costa E, Guidotti A. Clozapine and sulpiride but not haloperidol or olanzapine activate brain DNA demethylation. Proc Natl Acad Sci U S A. 2008;105: 13614–13619. 10.1073/pnas.0805493105 18757738 PMC2533238

[42] Kraepelin E. Dementia praecox and paraphrenia New York: Robert E. Krieger Publishing, 1971; 1919.

[43] Bleuler. Dementia praecox or the group of schizophrenias New York: International Universities Press, 1950; 1911.

[44] Archer T. Neurodegeneration in schizophrenia. Expert Rev Neurother. 2010;10: 1131–1141. 10.1586/ern.09.152 20586693

[45] Rund BR. Is schizophrenia a neurodegenerative disorder? Nord J Psychiatry. 2009;63: 196–201. 10.1080/08039480902767286 19235629

[46] Voineskos D, Rogasch NC, Rajji TK, Fitzgerald PB, Daskalakis ZJ. A review of evidence linking disrupted neural plasticity to schizophrenia. Can J Psychiatry Rev Can Psychiatr. 2013;58: 86–92. 10.1177/070674371305800205 23442895

[47] Yin D-M, Chen Y-J, Sathyamurthy A, Xiong W-C, Mei L. Synaptic dysfunction in schizophrenia. Adv Exp Med Biol. 2012;970: 493–516. 10.1007/978-3-7091-0932-8_22 22351070

[48] Balu DT, Coyle JT. Neuroplasticity signaling pathways linked to the pathophysiology of schizophrenia. Neurosci Biobehav Rev. 2011;35: 848–870. 10.1016/j.neubiorev.2010.10.005 20951727 PMC3005823

[49] Keefe RSE, Harvey PD. Cognitive impairment in schizophrenia. Handb Exp Pharmacol. 2012; 11–37. 10.1007/978-3-642-25758-2_2 23027411

[50] Park S, Holzman PS. Schizophrenics show spatial working memory deficits. Arch Gen Psychiatry. 1992;49: 975–982. 1449384 10.1001/archpsyc.1992.01820120063009

[51] Park S, Holzman PS. Association of working memory deficit and eye tracking dysfunction in schizophrenia. Schizophr Res. 1993;11: 55–61. 8297805 10.1016/0920-9964(93)90038-k

[52] Gross G, Huber G. [Schizophrenia: neurodevelopmental disorder or degenerative brain process?]. Fortschr Neurol Psychiatr. 2008;76 Suppl 1: S57–62. 10.1055/s-2008-1038153 18461546

[53] Bowie CR, Harvey PD. Treatment of cognitive deficits in schizophrenia. Curr Opin Investig Drugs Lond Engl 2000. 2006;7: 608–613. 16869112

[54] Psychiatrie A f M u D in d. Das AMDP-System: Manual zur Dokumentation psychiatrischer Befunde Auflage: 8., Aufl Hogrefe Verlag; 2006.

[55] Tinnes S, Ringwald J, Haas CA. TIMP-1 inhibits the proteolytic processing of Reelin in experimental epilepsy. FASEB J Off Publ Fed Am Soc Exp Biol. 2013;27: 2542–2552. 10.1096/fj.12-224899 23493620

[56] Fatemi SH, Reutiman TJ, Folsom TD. Chronic psychotropic drug treatment causes differential expression of Reelin signaling system in frontal cortex of rats. Schizophr Res. 2009;111: 138–152. 10.1016/j.schres.2009.03.002 19359144

[57] Underhill GH, George D, Bremer EG, Kansas GS. Gene expression profiling reveals a highly specialized genetic program of plasma cells. Blood. 2003;101: 4013–4021. 10.1182/blood-2002-08-2673 12543863

[58] Smalheiser NR, Costa E, Guidotti A, Impagnatiello F, Auta J, Lacor P, et al Expression of reelin in adult mammalian blood, liver, pituitary pars intermedia, and adrenal chromaffin cells. Proc Natl Acad Sci U S A. 2000;97: 1281–1286. 10655522 10.1073/pnas.97.3.1281 PMC15597

[59] Folsom TD, Fatemi SH. The involvement of Reelin in neurodevelopmental disorders. Neuropharmacology. 2013;68: 122–135. 10.1016/j.neuropharm.2012.08.015 22981949 PMC3632377

[60] Ignatova N, Sindic CJM, Goffinet AM. Characterization of the various forms of the Reelin protein in the cerebrospinal fluid of normal subjects and in neurological diseases. Neurobiol Dis. 2004;15: 326–330. 10.1016/j.nbd.2003.11.008 15006702

[61] Chameau P, Inta D, Vitalis T, Monyer H, Wadman WJ, van Hooft JA. The N-terminal region of reelin regulates postnatal dendritic maturation of cortical pyramidal neurons. Proc Natl Acad Sci U S A. 2009;106: 7227–7232. 10.1073/pnas.0810764106 19366679 PMC2678467

[62] Van der Velden L, van Hooft JA, Chameau P. Altered dendritic complexity affects firing properties of cortical layer 2/3 pyramidal neurons in mice lacking the 5-HT3A receptor. J Neurophysiol. 2012;108: 1521–1528. 10.1152/jn.00829.2011 22696545

[63] Smit-Rigter LA, Wadman WJ, van Hooft JA. Alterations in Apical Dendrite Bundling in the Somatosensory Cortex of 5-HT(3A) Receptor Knockout Mice. Front Neuroanat. 2011;5: 64 10.3389/fnana.2011.00064 22163214 PMC3233707

[64] Shariati GR, Ahangari G, Hossein-nezhad A, Asadi SM, Pooyafard F, Ahmadkhaniha HR. Expression changes of serotonin receptor gene subtype 5HT3a in peripheral blood mononuclear cells from schizophrenic patients treated with haloperidol and Olanzapin. Iran J Allergy Asthma Immunol. 2009;8: 135–139. doi:08.03/ijaai.135139 doi: 08.03/ijaai.135139 20124604

[65] Brummelte S, Galea LM, Devlin AM, Oberlander TF. Antidepressant use during pregnancy and serotonin transporter genotype (SLC6A4) affect newborn serum reelin levels. Dev Psychobiol. 2013;55: 518–529. 10.1002/dev.21056 22692766

