# Correction: Increased Blood-Reelin-Levels in First Episode Schizophrenia

**DOI:** 10.1371/journal.pone.0142247

**Published:** 2015-11-02

**Authors:** Tobias Hornig, Carola Haas, Lukas Sturm, Bernd Fiebich, Ludger Tebartz van Elst

Dr. Carola Haas should be listed as the second author, and her affiliation is: Department of Neurosurgery, Exp. Epilepsy Research, Albert-Ludwigs-University, Breisacher Strasse 64, 79106 Freiburg, Germany. The contributions of this author are as follows: Analyzed the data, contributed reagents/materials/analysis tools. The correct citation is: Hornig T, Haas C, Sturm L, Fiebich B, Tebartz van Elst L (2015) Increased Blood-Reelin-Levels in First Episode Schizophrenia. PLoS ONE 10(8): e0134671. doi:10.1371/journal.pone.0134671
