# Supplementary material for: Increased Blood-Reelin-Levels in First Episode Schizophrenia
Source: PLoS One. 2015 Aug 25;10(8):e0134671. doi: 10.1371/journal.pone.0134671 (PMC4549220; doi:10.1371/journal.pone.0134671)
Supplement: S1 Dataset — (PDF) [file pone.0134671.s001.pdf]

Group	Code	Age	Medication	Education	Fam. Schiz	Fam. Psych
1	RKeli001	35	2	2	0	0
1	RKeli002	35	3	3	0	0
1	RKeli003	30	1	3	0	0
1	RKeli004	30	2	2	0	1
1	RKeli005	29	3	2	0	1
1	RKeli006	26	3	3	0	1
1	RKeli008	22	3	2	0	1
1	RKeli009	22	3	3	0	0
1	RKeli010	33	3	3	0	0
1	RKeli011	30	3	3	0	0
1	RKeli012	27	2	1	0	1
1	RKeli013	24	3	1	0	1
1	RKeli014	24	2	2	1	1
1	RKeli016	22	2	3	0	1
1	RKeli017	22	3	3	0	1
1	RKeli018	21	3	2	0	0
1	RKeli019	20	3	1	0	1
1	RKeli020	20	3	2	0	1
2	RKeli021	32	0	1	0	0
2	RKeli022	31	0	2	0	0
2	RKeli023	30	0	3	0	0
2	RKeli024	29	0	1	0	0
2	Rkeli025	29	0	3	0	0
2	RKeli026	25	0	1	0	0
2	RKeli027	25	0	2	0	0
2	RKeli028	23	0	3	0	0
2	RKeli029	22	0	2	0	0
2	RKeli030	32	0	3	0	0
2	RKeli031	32	0	1	0	0
2	RKeli033	26	0	2	0	0
2	RKeli034	25	0	3	0	0
2	RKeli035	25	0	3	0	0
2	RKeli036	24	0	3	0	0
2	RKeli038	22	0	2	0	0
2	RKeli039	21	0	3	0	0
2	RKeli040	21	0	2	0	0

Group	Gender	Serum_ELI	ELI_Control	450kD WB	340kD WB	180kD WB
1	1	5,2	8,95	0,14	0,48	0,15
1	1	0,56	0,81	0,42	0,66	0,24
1	1	0,98	1,48	0,12	0,59	0,19
1	1	1,18	1,92	0,18	0,41	0,17
1	1	2,36	5,11	0,05	0,58	0,09
1	1	4,16	8,21	0,08	0,65	0,12
1	1	2,29	4,93	0,07	0,67	0,11
1	1	3,77	7,79	0,09	0,62	0,08
1	2	2,03	4,22	0,06	0,37	0,06
1	2	5,29	8,99	0,18	0,52	0,06
1	2	1,73	3,37	0,06	0,39	0,12
1	2	1,56	2,91	0,13	0,36	0,09
1	2	2,58	5,64	0,12	0,38	0,14
1	2	2,74	6,02	0,95	2,82	0
1	2	3,96	8,01	0,09	0,34	0,08
1	2	4,05	8,1	0,05	0,25	0,09
1	2	0	0	0	0	0
1	2	2,33	5,02	0,29	0,49	0,18
2	1	2,78	6,11	0,3	0,68	0,17
2	1	3,17	6,89	0,64	0,86	0,21
2	1	2,95	6,47	0,25	0,85	0,26
2	1	1,44	2,59	0,22	0,59	0,12
2	1	2,54	5,56	0,19	0,49	0,07
2	1	0	0	0	0	0
2	1	0,49	0,76	0,04	0,31	0,08
2	1	0	0	0	0	0
2	1	1,13	1,79	0,07	0,35	0,05
2	2	2,15	4,54	0,08	0,63	0,1
2	2	1,19	1,94	0,15	0,44	0,14
2	2	1,39	2,45	0,17	0,32	0,19
2	2	1,53	2,82	0,15	0,44	0,23
2	2	0	0	0	0	0
2	2	0,86	1,24	0,1	0,35	0,12
2	2	0	0	0	0	0
2	2	0	0	0	0	0
2	2	2,22	4,73	0,12	0,33	0,08

ELI=ELISA [ng/µl]   WB=Westernblot [OD]

Group	1=patients	2=healthy		
Medication	1=aripirazole	2=olanzapine	3=risperidone	0=no med
Education	1=low	2=middle	3=high	
Fam. History	0=no	1=yes		
Gender	1=f	2=m		
